# Skeletal muscle mass predicts the outcome of nivolumab treatment for non-small cell lung cancer

**DOI:** 10.1097/MD.0000000000019059

**Published:** 2020-02-14

**Authors:** Mariko Tsukagoshi, Takehiko Yokobori, Toshiki Yajima, Toshitaka Maeno, Kimihiro Shimizu, Akira Mogi, Kenichiro Araki, Norifumi Harimoto, Ken Shirabe, Kyoichi Kaira

**Affiliations:** a Department of Innovative Cancer Immunotherapy, Gunma University Graduate School of Medicine; b Division of Hepatobiliary and Pancreatic Surgery; c Division of General Thoracic Surgery, Integrative Center of General Surgery; d Division of Allergy and Respiratory Medicine, Integrative Center of Internal Medicine, Gunma University Hospital, Maebashi Gunma; e Department of Respiratory Medicine, Saitama Medical University International Medical Center, Hidaka Saitama, Japan.

**Keywords:** lung cancer, nivolumab, programmed death 1, sarcopenia, skeletal muscle mass

## Abstract

Nivolumab, a monoclonal antibody targeting programmed cell death-1, significantly prolongs survival for patients with advanced non-small-cell lung cancer (NSCLC). However, little is known about the value of predictive biomarkers. Hence, we investigated the impact of skeletal muscle (SM) mass loss on clinical outcomes in NSCLC patients undergoing nivolumab treatment. Thirty patients with histologically confirmed NSCLC treated with nivolumab were included in this study. Computed tomography was used to determine SM loss based on the SM index (SMI). The SMI is the cross-sectional area of the bilateral psoas muscles at the third lumbar vertebra, divided by height squared. The cut-off values were defined as 6.36 cm^2^/m^2^ for men and 3.92 cm^2^/m^2^ for women. Among the 30 patients, 13 (43%) had SM loss. There was no significant association between SM loss and immune-related adverse events. The SM loss group had undergone significantly more prior chemotherapy cycles (*P* = .04). SM loss was significantly associated with fewer nivolumab cycles (*P* = .01). No patients in the SM loss group achieved a partial response. Patients with SM loss had a significantly shorter progression-free survival period (*P* = .008) and median overall survival than those with normal SM mass (10 vs 25 months, respectively, *P* = .03). SM loss was an independent prognostic factor of poor survival. In conclusion, SM loss may be a predictive factor of poor outcomes in NSCLS patients undergoing nivolumab therapy.

## 1 Introduction

Lung cancer is the leading cause of cancer-related death worldwide, often because patients frequently present with advanced stages of the disease. Non-small-cell lung cancer (NSCLC) accounts for approximately 85% of all lung cancers,^[1]^ and systemic therapy is generally indicated for patients with advanced or metastatic NSCLC. Recently, immune checkpoint inhibitors, such as anti-programmed cell death-1 (PD-1) antibodies and anti-programmed cell death-ligand 1 (PD-L1) antibodies, have improved clinical outcomes for a subset of patients with advanced NSCLC.^[2–5]^ Nivolumab, a monoclonal antibody targeting PD-1, prolonged the overall survival (OS) of previously treated patients with advanced NSCLC compared to docetaxel in 2 independent phase III studies.^[2,3]^

Previous studies have demonstrated that the positive expression of PD-L1 on tumor cells was a significant biomarker to predict favorable outcomes of anti-PD-1 treatment.^[2,4]^ However, the role of PD-L1 expression as a predictive biomarker of the response to nivolumab treatment remains controversial.^[6]^ Although some patients fail to respond to nivolumab treatment despite positive PD-L1 expression, others benefit clinically regardless of negative PD-L1 expression. Although nivolumab has some benefits for advanced NSCLC patients, little is known regarding the value of predictive biomarkers in NSCLC with the use of nivolumab. Therefore, reliable biomarkers are necessary to predict the response to nivolumab treatment.

Sarcopenia is defined by the progressive and generalized loss of skeletal muscle (SM) mass and strength.^[7]^ Recently, sarcopenia was identified as a potential new predictor of morbidity and mortality after surgery for several cancers. Among patients with NSCLC, the incidence of sarcopenia is reportedly 47%,^[8]^ and the loss of SM mass is a significant contributor to morbidity and OS.^[9]^ However, the prognostic significance of SM mass in patients undergoing immunotherapy is unclear. Therefore, this study aimed to determine the correlation between the loss of SM mass and clinical outcomes of nivolumab for the treatment of advanced NSCLC.

## 2 Materials and methods

### 2.1 Patient population

The medical records of all patients with histologically confirmed NSCLC treated with nivolumab at the Gunma University Hospital between January 2016 and December 2017 were retrospectively reviewed. The inclusion criteria were stage IIIB/IV NSCLC or recurrent NSCLC, candidates for nivolumab treatment after initial chemotherapy, age ≥20 years, an Eastern Cooperative Oncology Group (ECOG) performance status of 0 to 2, and adequate hematologic, hepatic, and renal functions. The Ethics Committee of Gunma University Hospital approved the study protocol, and all clinical samples were used in accordance with institutional guidelines and the Declaration of Helsinki, after obtaining signed informed consent from all participants (approval no. 1404). The present study includes the patients’ information from our previous study.^[10,11]^

### 2.2 Treatment and data collection

Nivolumab was administered intravenously at a dose of 3 mg/kg every 2 weeks per the manufacturer's guidelines. The baseline clinical and demographic characteristics and treatment-related details of all patients were collected. Safety was assessed by evaluating the incidence of immune-related adverse events (irAEs).

The clinical response to nivolumab was evaluated by computed tomography (CT) and assessed by investigators per the RECIST criteria, version 1.1.^[12]^ Progression-free survival (PFS) was defined as the period from the date of initial nivolumab administration until the date of documented disease progression or death from any cause. OS was defined as the period from the date of initial nivolumab administration to the date of death from any cause.

### 2.3 Image analysis and definition of SM loss

SM loss was determined using the SM index (SMI). All patients underwent CT assessment within 30 days before receiving anti-PD-1 treatment. The cross-sectional area of the bilateral psoas muscles at the third lumbar vertebra (L3) was measured by manual tracing. The SMI was measured and a cut-off value was determined, in accordance with a previous report,^[13]^ as follows: SMI = cross-sectional area of the bilateral psoas muscles/height^2^ (cm^2^/m^2^). The SMI cut-off values were defined as 6.36 cm^2^/m^2^ for males and 3.92 cm^2^/m^2^ for females.^[13]^ SM loss was defined based on these cut-off values, and the patients were classified accordingly.

### 2.4 Immunohistochemistry

We investigated serial sections that consisted of the resected surgical specimens and needle biopsies. We obtained 26 serial sections for PD-L1 because the cancer part of each serial section was depleted during the process of cutting. All specimens were cut into 4-μm thick sections and were mounted onto glass slides. All sections were deparaffinized in xylene, rehydrated, and incubated with fresh 0.3% hydrogen peroxide for 30 minutes at room temperature to block endogenous peroxidase activity. After rehydration with a graded series of ethanol, the sections were then heated in boiled water and in Immunosaver (Nishin EM, Tokyo, Japan) at 98 to 100°C for 45 minutes, and PD-L1 was retrieved using Universal Heat Induced Epitope Retrieval (HIER) antigen retrieval reagent (Abcam, ab208572, Tokyo, Japan) at 120°C for 20 minutes in an autoclave. Nonspecific binding sites were blocked by incubation with Protein Block Serum-Free (Dako, Carpinteria, CA) for 30 minutes. The sections were then incubated with the primary antibody (diluted by Dako REAL antibody diluent) overnight at 4°C. PD-L1 (Cell signaling, E1L3N Rabbit mAb, 1:200 dilution) was used. The Histofine Simple Stain MAX-PO (Multi) Kit (Nichirei, Tokyo, Japan) was used as the secondary antibody. Chromogen 3,3-diaminobenzidine tetrahydrochloride was applied as a 0.02% solution in 50 mM ammonium acetate-citrate acid buffer (pH 6.0) containing 0.005% hydrogen peroxide. The sections were lightly counterstained with hematoxylin and mounted.

The tissue sections were evaluated by 2 independent evaluators who were blinded to the patient data. The expression of PD-L1 was classified using a semiquantitative scoring method: 1 = <1%, 2 = 1% to 5%, 3 = 5% to 10%, 4 = 10% to 50%, and 5 = >50% of cells were positive. Tumors with a score of greater than 3 were considered positive.^[14]^

### 2.5 Statistical analysis

Categorical variables were assessed using the Fisher exact test. The Mann–Whitney *U* test was used to analyze continuous variables. OS was computed using the Kaplan–Meier method and compared using the log-rank test. Prognostic factors were examined by univariate and multivariate analyses using Cox proportional hazard model. The results were considered statistically significant at *P* < .05, and all statistical analyses were performed using JMP Pro 14 software (SAS Institute, Cary, NC).

## 3 Results

### 3.1 Patient characteristics

The demographic characteristics of all 30 patients included in this study are presented in Table 1. There were 23 male (77%) and 7 female (23%) patients with a median age of 67 (range, 47–82) years. The median body mass index (BMI) was 21.9 (range, 18.0–27.4). Four patients (13%) had an ECOG performance status (PS) of 2. Twenty-four patients (80%) were diagnosed with adenocarcinoma. Five patients were diagnosed with stage III NSCLC and 11 with stage IV. Fourteen patients had recurrence after surgery.

**Table 1:**
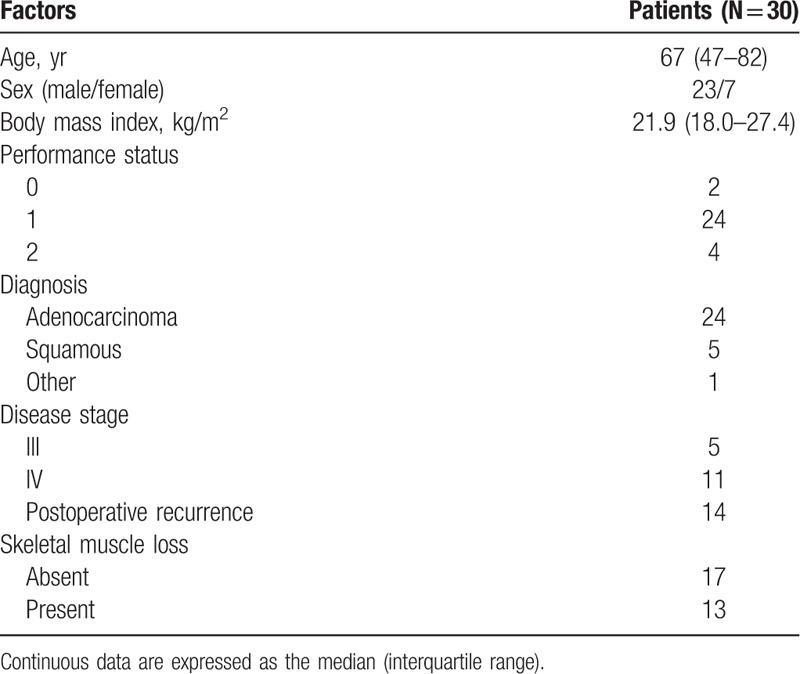
Patient characteristics.

Based on the SMI definition, 13 (43%) patients were allotted to the SM loss group and 17 (57%) to the normal SM mass group. Table 2 shows a comparison of the demographic and clinical characteristics between the 2 groups. There were no significant differences in age, sex, BMI, smoking status, or histology between the 2 groups. The SM loss group had undergone significantly more prior chemotherapy cycles (*P* = .04). No statistically significant associations were observed between SM loss and blood data of albumin, lymphocytes, and C-reactive protein levels. Additionally, no statistically significant association was observed between SM loss and irAEs (*P* = 1.00). Patients with SM loss received significantly fewer nivolumab cycles (*P* = .01).

**Table 2:**
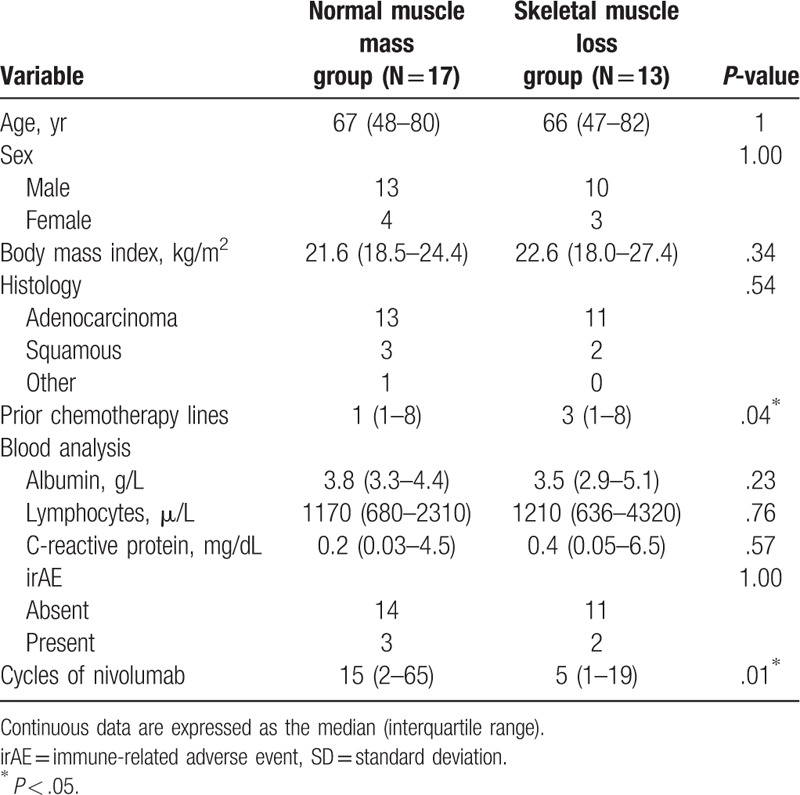
Clinical characteristics and skeletal muscle loss.

The association between the number of prior chemotherapy cycles and SM was investigated. Before nivolumab treatment, 17 patients were treated with 1 or 2 chemotherapy regimens, whereas 13 patients received 3 or more. SM loss was identified in 23.5% (4 of 17) of patients who had been treated with 1 or 2 chemotherapy regimens and in 69.2% (9 of 13) of patients who received 3 or more. SM loss was significant in patients who had been treated with 3 or more chemotherapy regimens (*P* = .02).

### 3.2 Association between SM loss and clinical response

Among the 30 patients, 6 (20%) achieved a partial response (PR). No patients in the SM loss group achieved a PR, demonstrating a significant association between SM loss and clinical response (*P* = .02) (Table 3). Furthermore, the association between the number of prior chemotherapy cycles and clinical response to nivolumab was investigated. Among the 6 patients who achieved a PR, 2 patients had been received 3 or more chemotherapy regimens before nivolumab treatment. There was no correlation between prior chemotherapy cycles and clinical response (*P* = .67).

**Table 3:**
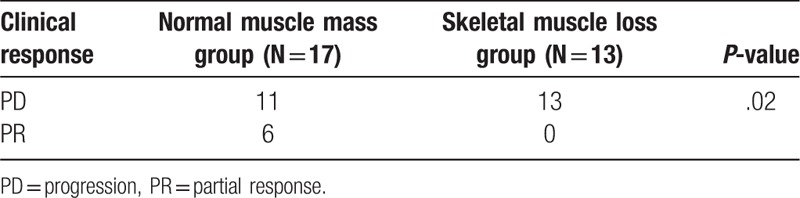
Clinical response and skeletal muscle loss.

### 3.3 Association between SM loss and prognosis

**Figure 1:**
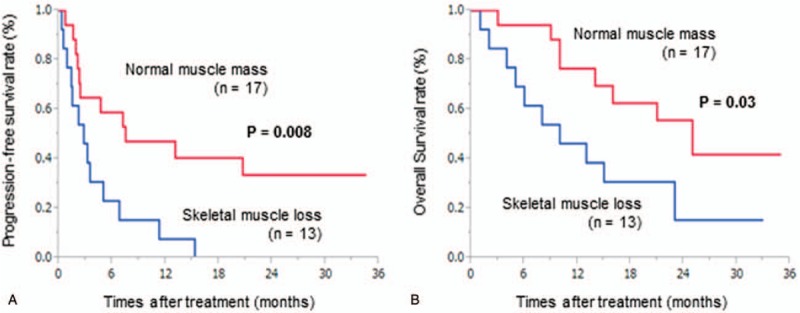
Kaplan–Meier plots showing PFS and OS according to SM mass. (A) PFS was significantly shorter (*P* = .008) among those with SM loss than among those with normal SM mass. (B) OS was significantly shorter (*P* = .03) among those with SM loss than among those with normal SM mass. OS = overall survival, PFS = progression-free survival, SM = skeletal muscle.

Figure 1 illustrates the prognostic significance of SM loss. Patients with SM loss had a significantly shorter median PFS than those with normal SM mass (2.8 vs 7.5 months, respectively, *P* = .008; Fig. 1A). Moreover, patients with SM loss had a significantly shorter median OS than those with normal SM mass (10 vs 25 months, respectively, *P* = .03; Fig. 1B).

### 3.4 Univariate and multivariate survival analyses

The univariate analysis revealed that smoking history (*P* = .02) and SM loss (*P* = .01) were significantly associated with PFS. The multivariate analysis revealed that smoking history and SM loss (risk ratio = 2.85; 95% confidence interval = 1.21–6.71; *P* = .02) were independent prognostic indicators of poor PFS (Table 4). Moreover, SM loss was significantly associated with OS (*P* = .04, Table 5).

**Table 4:**
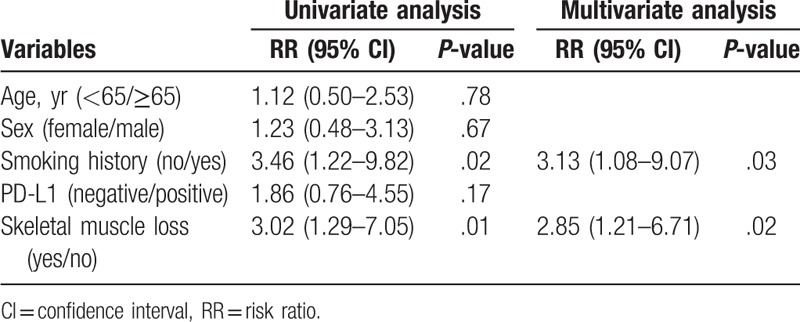
Univariate and multivariate analysis for progression-free survival.

**Table 5:**
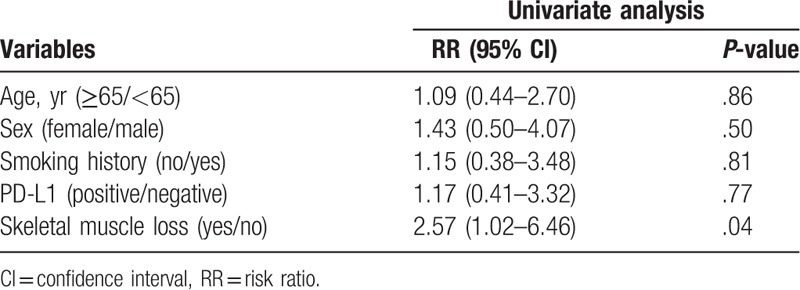
Univariate analysis for overall survival.

## 4 Discussion

This report focused on the impact of SM mass on the outcomes of NSCLC patients treated with nivolumab. The present study revealed, for the first time, significant associations between SM loss and shorter PFS and OS rates for patients with advanced NSCLC undergoing nivolumab treatment. SM loss was an independent prognostic factor of PFS. These findings emphasize the importance of assessing SM mass and identified SM loss as a predictive factor for nivolumab therapy.

Nivolumab treatment has improved the survival of patients with advanced NSCLC. However, relatively few patients fail to respond to this treatment regimen. Response to anti-PD-1 therapy, such as nivolumab, has been predicted by several internally consistent markers.^[15]^ The pre-therapy presence of CD8 tumor-infiltrating lymphocytes at the invasive tumor margin and PD-L1 expression are potential biomarkers of the response to anti-PD-1 therapy.^[16]^ In addition, tumors with a high mutation burden are more likely to achieve a clinical response.^[17]^ These findings occur because more neoantigens can lead to increased anti-PD-1 activity and may enhance the antitumor immune response.^[18–20]^ Furthermore, an interferon-γ gene signature is correlated with the response to anti-PD-1 therapy.^[21]^ For these reasons, the presence of tumor-reactive T cell infiltration is important for a response to anti-PD-1 therapy.

In the present study, the relevance of pre-therapy SM loss was investigated. Several recent studies investigating SM mass among transplantation patients associate SM loss with complications from post-transplantation infection.^[22]^ Some studies have revealed that SM loss is not only associated with an increased risk of postoperative bacterial infection but also a reduced risk of rejection compared to recipients with normal muscle mass post-transplantation.^[23]^ Further, low SM mass was an independent predictive factor for better graft survival.^[24]^ In the present study, there was no significant correlation between SM loss and irAEs. However, SM loss was significantly associated with fewer immunotherapy cycles since no patients in the SM loss group achieved a PR. These results indicate that SM mass might predict the response to immunotherapy with nivolumab. Moreover, there was a significant association between SM loss and the number of prior chemotherapy regimens in this study. SM loss was identified in approximately 70% of the patients after treatment with 3 or more chemotherapy regimens. These results suggest that a therapeutic effect might not be achieved even if the patient was treated with nivolumab after several chemotherapy cycles. Therefore, it might be necessary to consider starting nivolumab treatment before SM loss.

Okumura et al^[25]^ reported that decreasing muscle mass should produce fewer cytokines, which causes decreased immunity. The concept of SM as an endocrine organ that secretes cytokines, namely myokines, has recently received greater attention.^[26]^ Myokines can be defined as proteins, including interleukin (IL)-6, IL-15, IL-8, tumor necrosis factor-alpha, myostatin, irisin, and fibroblast growth factor 21, that are synthesized by SM tissue and exert either paracrine or autocrine effects.^[27]^ Lutz et al reported that decreased myokine levels suppress the function of natural killer cells in sarcopenia.^[28]^ IL-6, which is a major myokine, has many effects on the immune system and modulates multiple immune responses. Although generally regarded as a pro-inflammatory cytokine, IL-6 also has anti-inflammatory effects and can induce an anti-inflammatory environment.^[28,29]^ Recently, Cortellini et al^[30]^ reported the predictive value of SM mass in NSCLC patients treated with second-line nivolumab. Despite no statistically significant differences were observed, the median PFS and OS appear decidedly longer among patients with non-low SM mass compared to those with low SM mass. In the present study, SM loss was significantly associated with a shorter PFS and OS than was normal SM mass. Although the underlying molecular mechanism of the relationship between SM mass and immunotherapy remains unknown, this finding should help clarify the role of SM, including myokines, on immunotherapy.

Several studies have indicated the effect of amino acids on immune function. Nutritional status affects immune cell metabolism and function, and undernutrition has been associated with immunosuppression.^[31]^ Catabolized muscle proteins provide amino acids for protein synthesis for the immune response. In addition, an adequate supply of key amino acids is necessary to support efficient immune function.^[32]^ An enhanced supply of branched-chain amino acids and increased SM mass might improve nivolumab treatment outcomes.

There are several limitations to the present study. First, this was an observational cohort study. Second, the sample size was small. Third, we could not collect data on grip strength as an indicator of SM strength as a sarcopenia parameter. Fourth, SM loss might have been correlated with chemotherapy resistance because the SM loss group had undergone significantly more prior chemotherapy cycles. Hence, further studies with larger sample sizes are necessary to confirm and update our conclusions.

In conclusion, this is the first study to show that SM loss is closely correlated with shorter PFS and OS in NSCLC patients after nivolumab treatment. The evaluation of SM mass may be a useful surrogate marker of the treatment response and can help predict the clinical outcomes of patients receiving nivolumab therapy.

## Author contributions

**Conceptualization:** Mariko Tsukagoshi, Takehiko Yokobori, Toshiki Yajima, Ken Shirabe, Kyoichi Kaira.

**Data curation:** Mariko Tsukagoshi, Toshiki Yajima, Toshitaka Maeno, Kimihiro Shimizu, Akira Mogi, Kyoichi Kaira.

**Investigation:** Mariko Tsukagoshi, Kenichiro Araki, Norifumi Harimoto, Kyoichi Kaira.

**Methodology:** Takehiko Yokobori, Toshiki Yajima, Kenichiro Araki, Norifumi Harimoto, Kyoichi Kaira.

**Project administration:** Takehiko Yokobori, Toshiki Yajima, Ken Shirabe, Kyoichi Kaira.

**Supervision:** Toshitaka Maeno, Kimihiro Shimizu, Akira Mogi, Ken Shirabe, Kyoichi Kaira.

**Writing – original draft:** Mariko Tsukagoshi

**Writing – review & editing:** Mariko Tsukagoshi, Ken Shirabe, Kyoichi Kaira.

Mariko Tsukagoshi orcid: 0000-0003-3748-9615.
